# CH_4_ Adsorption Probability on GaN(0001) and (000−1) during Metalorganic Vapor Phase Epitaxy and Its Relationship to Carbon Contamination in the Films

**DOI:** 10.3390/ma12060972

**Published:** 2019-03-23

**Authors:** Akira Kusaba, Guanchen Li, Pawel Kempisty, Michael R. von Spakovsky, Yoshihiro Kangawa

**Affiliations:** 1 Department of Aeronautics and Astronautics, Kyushu University, Nishi-ku, Fukuoka 819-0395, Japan; 2 Department of Engineering Science, University of Oxford, Parks Road, Oxford OX1 3PJ, UK; guanchen.li@eng.ox.ac.uk; 3 Institute of High Pressure Physics, Polish Academy of Sciences, Sokolowska 29/37, 01-142 Warsaw, Poland; kempes@unipress.waw.pl; 4 Research Institute for Applied Mechanics, Kyushu University, Kasuga, Fukuoka 816-8580, Japan; kangawa@riam.kyushu-u.ac.jp; 5 Center for Energy Systems Research, Department of Mechanical Engineering, Virginia Tech, Blacksburg, VA 24061, USA; vonspako@vt.edu; 6 Center for Integrated Research of Future Electronics, Institute of Materials and Systems for Sustainability, Nagoya University, Chikusa-ku, Nagoya 464-8603, Japan

**Keywords:** metalorganic vapor phase epitaxy, gallium nitride, density functional theory calculations, steepest-entropy-ascent quantum thermodynamics

## Abstract

Suppression of carbon contamination in GaN films grown using metalorganic vapor phase epitaxy (MOVPE) is a crucial issue in its application to high power and high frequency electronic devices. To know how to reduce the C concentration in the films, a sequential analysis based on first principles calculations is performed. Thus, surface reconstruction and the adsorption of the CH_4_ produced by the decomposition of the Ga source, Ga(CH_3_)_3_, and its incorporation into the GaN sub-surface layers are investigated. In this sequential analysis, the dataset of the adsorption probability of CH_4_ on reconstructed surfaces is indispensable, as is the energy of the C impurity in the GaN sub-surface layers. The C adsorption probability is obtained based on steepest-entropy-ascent quantum thermodynamics (SEAQT). SEAQT is a thermodynamic ensemble-based, non-phenomenological framework that can predict the behavior of non-equilibrium processes, even those far from equilibrium. This framework is suitable especially when one studies the adsorption behavior of an impurity molecule because the conventional approach, the chemical potential control method, cannot be applied to a quantitative analysis for such a system. The proposed sequential model successfully explains the influence of the growth orientation, GaN(0001) and (000−1), on the incorporation of C into the film. This model can contribute to the suppression of the C contamination in GaN MOVPE.

## 1. Introduction

GaN is a promising material for the next generation of semiconductor power devices [1]. A vertical GaN power device beyond 1 kV operation, which requires a thick (more than 10 μm) drift layer with a C concentration less than 10^16^ cm^−3^, is suitable for the control of the main motor of a hybrid vehicle (HV) or an electric vehicle (EV) [2]. The drift layer (i.e., epitaxial GaN film) for the device is grown using metalorganic vapor phase epitaxy (MOVPE). In the GaN MOVPE system, Ga(CH_3_)_3_ (trimethylgallium, TMG) is used as the source gas of a group-III element (Ga) and is also the source of an unintentional C impurity. To reduce the manufacturing cost of the GaN power device with the thick drift layer, the high growth rate MOVPE technology is needed. However, the increase in TMG input has a negative impact on the C impurity contamination. For this reason, the theoretical prediction of the C concentration is important. According to thermodynamic analyses, activation energy calculations [3], and time-of-flight (TOF) high-resolution and high-sensitivity mass spectrometry measurements [4], TMG decomposes via reacting with the H_2_ carrier gas and/or nitrogen source (NH_3_), producing CH_4_. Therefore, the interaction between the CH_4_ molecule and the GaN surface during the MOVPE growth process must be understood clearly from the standpoint of reducing the unintentional C impurity contamination. It is worth noting that the chemical potential control method is not able to quantitatively explain the differences in the incorporation of impurities at different crystallographic orientations. This is due to the fact that the gaseous chemical potential is a macroscopic property, whose mean value is calculated based on the distribution of gaseous microscopic states. For example, for the case of a Maxwell distribution, most of molecules are near the peak of the distribution, which means that the molecules near the peak are reflected strongly in the mean value (i.e., the translational energy of the molecule) and the molecules far from the peak are not. This is true in a similar vein for the chemical potential. Thus, the chemical potential control method can explain whether or not most of the molecules are deposited on the surface and is, thus, adequate for describing the deposition of material molecules (e.g., Ga monoatomic molecule after TMG decomposition). However, it is unable to account for the minor part of the CH_4_ molecules, which have values far from the mean. This small number of CH_4_ molecules can influence the GaN power device performance via C impurities. In fact, References [5,6] indicate that the electron mobility of GaN films is strongly influenced by C impurities. For a quantitative analysis of the adsorption behavior of such an impurity, steepest-entropy-ascent quantum thermodynamics (SEAQT) is suitable because the expression of a state in SEAQT is based on the distribution itself and has the information of the minor part. In addition, the surface structure, on which the CH_4_ is adsorbed, and the stability of the C impurity in the sub-surface layers should be considered for the quantitative prediction of the C concentration in GaN films. In this study, this sequential analysis (surface reconstruction, CH_4_ adsorption, and C incorporation) was carried out to understand the influence of growth orientation, GaN(0001) and (000−1), on the C concentration in GaN films. First, the GaN surface reconstructions during MOVPE are determined using an approach that compares surface formation energies. (Section 2.1). For these reconstructed surfaces, CH_4_ adsorption structures are then studied using a density functional theory (DFT) total energies comparison (Section 2.2). In addition, the stabilization of the adsorption structures is discussed based on an adsorption free energies comparison. Then, based on SEAQT, Section 3 describes the non-equilibrium adsorption process and adsorption probability for the adsorption structure revealed in Section 2.2. Finally, Section 4 provides the model for the growth orientation dependence of the C concentration using the adsorption probability obtained in Section 3.

## 2. Adsorption Structure

### 2.1. Reconstructed Surface During the MOVPE Process

**Figure 1:**
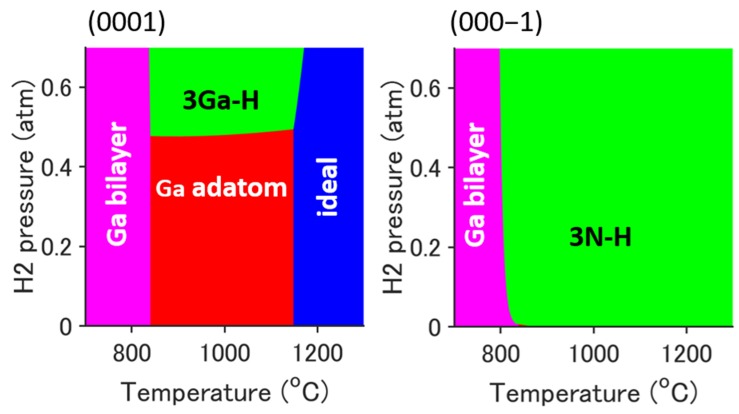
Surface phase diagram of GaN(0001) and (000−1) during the MOVPE growth process. The growth conditions are *p*_Ga_ = 2.5 × 10^−4^ atm and *p*_carrier_ = *p*_N2_ + *p*_H2_ = 0.7 atm.

It is well known that various reconstructed surfaces appear depending on the temperature and partial pressures, which occurs during the growth process. In 2001, Kangawa et al. proposed a theoretical approach for creating a surface phase diagram using the chemical potentials of molecules as functions of the temperature and partial pressures [7]. To date, reconstructed structures for polar, non-polar, and semi-polar GaN surfaces during the growth process have been studied [8,9,10,11,12,13,14,15,16,17,18,19]. Kusaba et al. reported the Ga adatom surface and the 3Ga-H surface for (0001) and the 3N-H surface for (000−1) appear for typical GaN MOVPE conditions [19]. Thus, the influence of the H_2_ partial pressure on surface phase transition can be provided. The chemical potential of a molecule μ is given by:

(1) $$\mu =-k_{B}T\ln\left(\left(gk_{B}T/p\right)\zeta_{\mathrm{trans}}\zeta_{\mathrm{rot}}\zeta_{\mathrm{vibr}}\right)$$

(2) $$\zeta_{\mathrm{trans}}={\left(2\pi mk_{B}T/h^{2}\right)}^{3/2}$$

(3) $$\zeta_{\mathrm{rot}}=\left(1/\pi \sigma \right){\left\{8\pi^{3}{\left(I_{A}I_{B}\cdots \right)}^{1/n}k_{B}T/h^{2}\right\}}^{n/2}$$

(4) $$\zeta_{\mathrm{vibr}}=\overset{3N-3-n}{\underset{i}{\prod }}{\left\{1-\exp\left(-h\nu_{i}/k_{B}T\right)\right\}}^{-1}$$

where $\zeta_{\mathrm{trans}}$, $\zeta_{\mathrm{rot}}$, and $\zeta_{\mathrm{vibr}}$ are the partition functions for the translational, rotational, and vibrational motion, respectively. Here, $k_{B}$ is Boltzmann’s constant, T the gas temperature, g the degeneracy of the electron energy level, p the partial pressure of the particle, m the mass of one particle, h Planck’s constant, σ the symmetry factor, $I_{I}$ the moment of inertia, n the number of rotational degrees of freedom, N the number of atoms in the particle, i the degree of freedom for vibration, and ν the frequency [7]. The formation energy of a reconstructed surface is given by:

(5) $$E_{f}\left(T,p_{\mathrm{Ga}},p_{N2},p_{H2}\right)=E_{\mathrm{ad}}-n_{\mathrm{Ga}}\mu_{\mathrm{Ga}}\left(T,p_{\mathrm{Ga}}\right)-n_{N}\mu_{N2}\left(T,p_{N2}\right)/2-n_{H}\mu_{H2}\left(T,p_{H2}\right)/2,$$

(6) $$E_{\mathrm{ad}}=E_{\mathrm{recon}}-\left(E_{\mathrm{ideal}}+n_{\mathrm{Ga}}E_{\mathrm{Ga}}+n_{N}E_{N2}/2+n_{H}E_{H2}/2\right),$$

where $E_{\mathrm{ad}}$ is the adsorption energy; $E_{\mathrm{recon}}$, $E_{\mathrm{ideal}}$, $E_{\mathrm{Ga}}$, $E_{N2}$, and $E_{H2}$ are the total energies of the reconstructed surface, ideal surface, Ga molecule, N_2_ molecule, and H_2_ molecule, respectively; and $n_{\mathrm{Ga}}$, $n_{N}$, and $n_{H}$ are the numbers of excess atoms of the reconstructed surface. The stable surface, which has the minimum formation energy among the possible candidates, is shown on the surface phase diagram. Figure 1 shows the dependence of the H_2_ partial pressure on surface reconstruction. The growth conditions are set as follows:$\;p_{\mathrm{Ga}}=2.5\times 10^{-4}\;\mathrm{atm}$ and $p_{\mathrm{carrier}}=p_{N2}+p_{H2}=0.7\;\mathrm{atm}$, which means that the V/III ratio, $p_{\mathrm{NH}3}/p_{\mathrm{Ga}}$, is set at 1200. The details of the structures of candidate reconstructed surfaces are provided in Reference [19]. In the case of GaN(0001) growth, a hydrogen adsorbed surface, 3Ga-H (2 × 2), appears at a H_2_ partial pressure greater than about 0.5 atm and around a typical growth temperature of 1050 °C. In the case of GaN(000−1) growth, a hydrogen adsorbed surface, 3N-H (2 × 2), appears in the H_2_ partial pressure range of about 0.0 atm to 0.7 atm above 800 °C . Thus, these growth surfaces (i.e., surface reconstructions: 3Ga-H and 3N-H surfaces) are assumed hereafter.

### 2.2. Adsorption Structure and Its Stabilization

**Figure 2:**
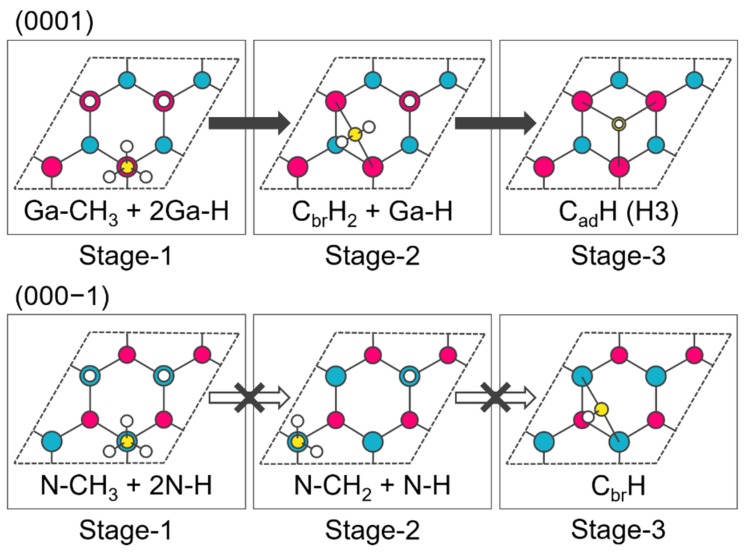
Adsorption structures of CH_4_ onto the 3Ga-H and 3N-H (2 × 2) surfaces. Red atoms represent Ga, blue atoms N, yellow atoms C, and white atoms H. Stage-1, Stage-2, and Stage-3 show the structures after the desorption of one, two, and three H_2_ molecules, respectively.

The structures after the adsorption of one CH_4_ molecule and the desorption of one H_2_ molecule onto/from 3Ga-H (3N-H) are designated as Stage-1, while those after the desorption of an additional one H_2_ molecule and two H_2_ molecules are designated as Stage-2 and Stage-3, respectively. The candidate structures are constructed on the basis of the electron counting (EC) rule [20]. Figure 2 shows the stable structures determined by DFT energetics for each stage. All electron calculations are made using the DMol^3^ software package [21,22] with the Perdew–Burke–Ernzerhof (PBE) functional [23] and the double numerical plus polarization (DNP) basis set. The calculated 2 × 2 slab models comprise a vacuum layer of more than 20 Å and five GaN bilayers whose bottom layer is fixed and passivated with fictitious hydrogen atoms [24]. A basis set cutoff of 4.8 Å and a 3 × 3 × 1 Monkhorst-Pack (MP) k-point mesh [25] are used. The geometry optimization convergence thresholds are 2.0 × 10^−5^ Ha, 0.0005 Ha/Å, and 0.005 Å for the energy change, maximum force, and maximum displacement, respectively. Ga-CH_3_ and N-CH_3_ are the first adsorption structures of CH_4_ and these structures are constructed by the reaction between the H atom of CH_4_ and the adsorbed H atom of 3Ga-H (3N-H) producing H_2_. Comparing Stage-2 structures, the stable structure in (000−1) is not a bridge structure like (0001). This implies that the strained bridge structure in (000−1) is unstable because of the short N–C bond length. In Stage-3, the (000−1) structure is different from the (0001) structure because of a similar reason. 

**Figure 3:**
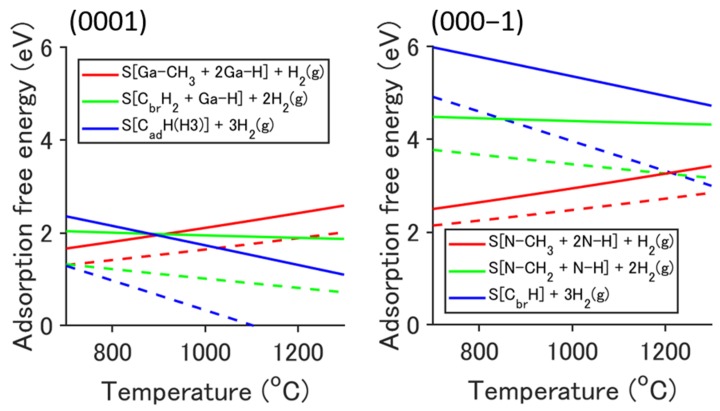
Adsorption free energy of CH_4_ onto the 3Ga-H and 3N-H (2 × 2) surfaces. The red, green, and blue lines correspond to Stage-1, Stage-2, and Stage-3 in Figure 2, respectively. The growth conditions are *p*_CH4_ = 3*p*_Ga_ = 7.5 × 10^−4^ atm, *p*_H2_ = 0.7 atm (solid lines), and *p*_H2_ = 0.01 atm (dashed lines).

The stability of the structures of Stage-1, Stage-2, and Stage-3 are compared on a thermodynamic equilibrium basis. The adsorption free energy of CH_4_ onto the 3Ga-H and 3N-H (2 × 2) surfaces is given by:

(7) $$E_{f}\left(T,p_{\mathrm{CH}4},p_{H2}\right)=E_{\mathrm{ad}}+n_{H2}\mu_{H2}\left(T,p_{H2}\right)-\mu_{\mathrm{CH}4}\left(T,p_{\mathrm{CH}4}\right)$$

(8) $$E_{\mathrm{ad}}=\left(E_{C\_\mathrm{ad}}+n_{H2}E_{H2}\right)-\left(E_{3H}+E_{\mathrm{CH}4}\right),$$

where $E_{C\_\mathrm{ad}}$, $E_{3H},$ and $E_{\mathrm{CH}4}$ are the total energies of the CH_4_ adsorbed surface, the 3Ga-H (3N-H) surface, and the CH_4_ molecule, respectively. $n_{H2}$ is the number of desorbed H_2_ molecules, and is equal to 1 for Stage-1, 2 for Stage-2, and 3 for Stage-3. Figure 3 shows the adsorption free energy for the case of the H_2_ carrier gas ($p_{H2}=0.7\;\mathrm{atm}$, solid lines) and the N_2_ carrier gas ($p_{H2}=0.01\;\mathrm{atm}$, dashed lines). In (0001), it is suggested that thermodynamically, the stabilization of the CH_4_ adsorbed structure at typical growth temperatures, ≈1000 °C, proceeds as follows: S[Ga-CH_3_ + 2Ga-H] + H_2_(g) → S[C_br_H2 + Ga-H] + 2H_2_(g) → S[C_ad_H(H3)] + 3H_2_(g) (see Stage-1, Stage-2, and Stage-3 in Figure 2, respectively). At temperatures less than about 900 °C, this stabilization would not occur in the case of the H_2_ carrier gas. In (000−1), the structures of Stage-2 and Stage-3 are much more unstable than the structure of Stage-1. Thus, the adsorption structure would not change from Stage-1 regardless of the H_2_ partial pressure.

## 3. Adsorption Probability

### 3.1. Steepest-Entropy-Ascent Quantum Thermodynamics

SEAQT is a thermodynamic-ensemble-based, non-phenomenological framework that can predict the behavior of non-equilibrium processes, even those far from equilibrium. This framework has been developed and applied to both non-reacting and reacting systems at multiple spatial and temporal scales and validated via comparisons with experiments [26,27,28,29,30,31,32,33,34,35,36,37,38,39,40,41,42,43,44,45,46,47]. In 2016, Li and von Spakovsky developed a density of states method and introduced the concept of a hypoequilibrium state, which extends the computational applicability of this framework across all spatial and temporal scales [41]. In the SEAQT framework, a thermodynamic state is defined via a probability distribution $\left\{p_{i}\right\}$ (or in strictly quantum mechanical terms, a density operator) among the quantum mechanical energy eigenlevels $\left\{\epsilon_{i}\right\}$, which may have degeneracy $\left\{n_{i}\right\}$. A thermodynamic property is defined as the ensemble average [48,49] such that for the energy and entropy:

(9) $$E=<e>=\underset{i}{\sum }p_{i}\epsilon_{i}$$

(10) $$S=<s>=\underset{i}{\sum }-p_{i}\ln\left(\frac{p_{i}}{n_{i}}\right)$$

The time evolution of the system state, $\left\{p_{i}\right\}$, is predicted using the SEAQT equation of motion, which is comprised of a reversible and an irreversible dynamics. The reversible dynamics describes the mechanics based on the time-dependent part of the Schrödinger (or equivalent von Neumann) equation, while the irreversible dynamics, captured via the principle of steepest entropy ascent (SEA) [26,27], depicts the intrinsic entropy generation required by the second law of thermodynamics. According to this principle, the irreversible relaxation is in the direction that has the largest entropy gradient consistent with the laws for the conservation of mass and energy, i.e., in this case, $\underset{i}{\sum }p_{i}=constant$ and E=constant. For a dilute Boltzmann gas and no correlations, the reversible dynamics disappears, and the equation of motion for the case when the only generators of the motion are the identity and Hamiltonian operators^1^ reduces to:

(11) $$\frac{dp_{i}}{dt}=\frac{1}{\tau }\frac{\left|\begin{array}{ccc} -p_{i}\ln\left(\frac{p_{i}}{n_{i}}\right) & p_{i} & p_{i}\epsilon_{i}\\ <s> & 1 & <e>\\ <es> & <e> & <e^{2}> \end{array}\right|}{\left|\begin{array}{cc} 1 & <e>\\ <e> & <e^{2}> \end{array}\right|},$$

^1^ Note that the basis for the formulation of the SEAQT equation of motion is one, which uses the quantum mechanical operator format, so that the number and types of generators of the motion, i.e., quantum mechanical operators, used to derive this equation vary according to the type of system and types of phenomena being modeled. Thus, Equation (11) as written is specific to the case at hand.

where τ is the relaxation time and the ensemble averages appearing in this equation are given by:

(12) $$<es>=\underset{i}{\sum }-p_{i}\epsilon_{i}\ln\left(\frac{p_{i}}{n_{i}}\right)$$

(13) $$<e^{2}>=\underset{i}{\sum }p_{i}\epsilon_{i}^{2}$$

Although τ is strictly speaking a functional of the state’s probability distribution, it can be and is often treated as a constant. For arbitrary values of τ, Equation (11) uniquely predicts the kinetics but not the actual dynamics of the state evolution for a given initial state [41]. To correlate the kinetics to the actual time with which the state evolution proceeds along the kinetic path, i.e., to the dynamics, τ can be determined via comparisons with experimental data [32,35,38,46] or from *ab initio* calculations based on quantum or classical mechanical considerations [30,37,42,47]. For details of the derivation of the SEAQT equation of motion, the reader is referred to References [26,27,28,29,30,41].

### 3.2. System Definition and Calculation Condition

In Section 2, the stabilization of the adsorption structures (i.e., Stage-1 → Stage-2 → Stage-3) was discussed qualitatively using an adsorption free energies comparison even though the reaction in (000−1) cannot proceed to Stage-2. In this section, the amount of CH_4_ adsorption (i.e., 3Ga-H (3N-H) → Stage-1) is discussed quantitatively using the SEAQT framework to theoretically predict the concentration of C in a GaN film. Doing so requires knowing the growth orientation dependence of the adsorption probability. The reaction mechanism for GaN(0001) and (000−1) considered is as follows:

(14) $${\mathrm{CH}}_{4}\left(g\right)+S1\to H_{2}\left(g\right)+S2,$$

where S1 denotes S[3Ga-H] and S[3N-H], and S2 denotes ${S[\mathrm{Ga}-\mathrm{CH}}_{3}+2\mathrm{Ga}-H]$ and ${S[N-\mathrm{CH}}_{3}+2N-H]$ in (0001) and (000−1), respectively. Subsystem 1 (i.e., the reactants) is comprised of one CH_4_ molecule and a 2 × 2 S1 surface (i.e., three H adatoms), subsystem 2 (i.e., the products) is comprised of one H_2_ molecule and a 2 × 2 S2 surface (i.e., one CH_3_ admolecule and two H adatoms). In 2017, Kusaba et al. proposed the SEAQT chemical adsorption model at the semiconductor surface coupled with DFT calculations [50]. In the paper, the excited states of the vibrational modes of the adsorbates were neglected for simplification. In the present study, these excited states were taken into account for a more accurate analysis. Therefore, the energy eigenlevels of the eigenstructures for subsystems 1 and 2 (i.e., $\left\{\epsilon_{i}^{\mathrm{sub}1}\right\}$ and $\left\{\epsilon_{i}^{\mathrm{sub}2}\right\}$) are given by:

(15) $$\epsilon_{i}^{\mathrm{sub}1}=E_{\mathrm{DFT}}^{\mathrm{CH}4}+E_{\mathrm{DFT}}^{S1}+E_{\mathrm{ZPV}}^{\mathrm{CH}4}+E_{\mathrm{ZPV}}^{\mathrm{ad}1}+\epsilon_{i}^{\mathrm{CH}4+\mathrm{ad}1}$$

(16) $$\epsilon_{i}^{\mathrm{sub}2}=E_{\mathrm{DFT}}^{H2}+E_{\mathrm{DFT}}^{S2}+E_{\mathrm{ZPV}}^{H2}+E_{\mathrm{ZPV}}^{\mathrm{ad}2}+\epsilon_{i}^{H2+\mathrm{ad}2},$$

where i is the index of the energy eigenlevel; $E_{\mathrm{DFT}}^{\mathrm{CH}4}$, $E_{\mathrm{DFT}}^{H2}$, $E_{\mathrm{DFT}}^{S1}$, and $E_{\mathrm{DFT}}^{S2}$ are the total energies of the CH_4_ and H_2_ molecules and the surface slab models of S1 and S2 determined using DFT calculations, respectively; $E_{\mathrm{ZPV}}^{\mathrm{CH}4}$, $E_{\mathrm{ZPV}}^{H2}$, $E_{\mathrm{ZPV}}^{\mathrm{ad}1}$, and $E_{\mathrm{ZPV}}^{\mathrm{ad}2}$ are the zero-point energies of the CH_4_ and H_2_ molecules and the adsorbates of S1 and S2, respectively; the $\left\{\epsilon_{i}^{\mathrm{CH}4+\mathrm{ad}1}\right\}$ are the joint energy eigenlevels of the CH_4_ molecule and the adsorbates of S1; and the $\left\{\epsilon_{i}^{H2+\mathrm{ad}2}\right\}$ are those of the H_2_ molecule and the adsorbates of S2. Note that adsorbates only have a vibrational mode, while gaseous molecules have translational, rotational, and vibrational modes. These joint energy eigenlevels are constructed from the translational density of states (DOS), the rotational DOS, and the vibrational discrete levels, given as:

(17) $$D_{\mathrm{tra}}\left(\epsilon_{\mathrm{tra}}\right)=\frac{2\pi V}{h^{3}}{\left(2m\right)}^{\frac{3}{2}}\epsilon_{\mathrm{tra}}{}^{\frac{1}{2}}$$

(18) $$B=\frac{h^{2}}{8\pi^{2}I}$$

(19) $$D_{\mathrm{rot}}^{\mathrm{spherical}}\left(\epsilon_{\mathrm{rot}}\right)=\frac{2}{\sigma B^{\frac{3}{2}}}\epsilon_{\mathrm{rot}}{}^{\frac{1}{2}}$$

(20) $$D_{\mathrm{rot}}^{\mathrm{linear}}\left(\epsilon_{\mathrm{rot}}\right)=\frac{1}{\sigma B}$$

(21) $$\epsilon_{\mathrm{vib}}=Lh\nu_{M}$$

where h is Planck’s constant, V is the volume (set equal to 0.001 m^3^), m is the particle mass, I is the moment of inertia, σ is the symmetry factor (2 for H_2_, 12 for CH_4_), L is the quantum number (0, 1, 2, …), and $\nu_{M}$ is the vibrational frequency of the *M*-th mode. The translational and rotational DOS is constructed as follows [41]:

(22) $$\begin{array}{cc} D_{\mathrm{tra},\mathrm{rot}}^{\mathrm{CH}4}\left(E\right)dE & =\int_{\epsilon_{\mathrm{tra}}^{\mathrm{CH}4}+\epsilon_{\mathrm{rot}}^{\mathrm{CH}4}=E}D_{\mathrm{tra}}^{\mathrm{CH}4}\left(\epsilon_{\mathrm{tra}}^{\mathrm{CH}4}\right)D_{\mathrm{rot}}^{\mathrm{CH}4}\left(\epsilon_{\mathrm{rot}}^{\mathrm{CH}4}\right)d\epsilon_{\mathrm{tra}}^{\mathrm{CH}4}d\epsilon_{\mathrm{rot}}^{\mathrm{CH}4}\\ & =\frac{2\pi V}{h^{3}}{\left(2m_{\mathrm{CH}4}\right)}^{\frac{3}{2}}\frac{2}{\sigma_{\mathrm{CH}4}B_{\mathrm{CH}4}{}^{\frac{3}{2}}}Β\mathrm{eta}\left(\frac{3}{2},\;\frac{3}{2}\right)E^{2}dE \end{array}$$

(23) $$\begin{array}{cc} D_{\mathrm{tra},\mathrm{rot}}^{H2}\left(E\right)dE & =\int_{\epsilon_{\mathrm{tra}}^{H2}+\epsilon_{\mathrm{rot}}^{H2}=E}D_{\mathrm{tra}}^{H2}\left(\epsilon_{\mathrm{tra}}^{H2}\right)D_{\mathrm{rot}}^{H2}\left(\epsilon_{\mathrm{rot}}^{H2}\right)d\epsilon_{\mathrm{tra}}^{H2}d\epsilon_{\mathrm{rot}}^{H2}\\ & =\frac{2\pi V}{h^{3}}{\left(2m_{H2}\right)}^{\frac{3}{2}}\frac{1}{\sigma_{H2}B_{H2}}Β\mathrm{eta}\left(\frac{3}{2},\;1\right)E^{\frac{3}{2}}dE \end{array}$$

where Βeta( , ) denotes the beta function. Furthermore, $D_{\mathrm{tra},\mathrm{rot}}\left(E\right)$ is discretized to generate the energy pseudo-eigenstructure $\left\{\epsilon_{i}^{\mathrm{tra},\mathrm{rot}}\right\}$, $\left\{n_{i}^{\mathrm{tra},\mathrm{rot}}\right\}$ (see Reference [41]). This translational and rotational energy pseudo-eigenstructure is then joined with the vibrational eigenstructures of the molecules and adsorbates. The degeneracies are determined as follow:

(24) $$n_{i}^{\mathrm{tra},\mathrm{rot},\mathrm{Mvib}}=\sum_{L}n_{i-\left(\mathrm{Lh}\nu \_M\right)/\Delta }^{\mathrm{tra},\mathrm{rot},(M-1)\mathrm{vib}}$$

where $\left(Lh\nu_{M}\right)/\Delta$ is the number of index i for $\epsilon_{i}^{\mathrm{tra},\mathrm{rot}}<Lh\nu_{M}$. The procedure of Equation (24) is repeated for all *M* (i.e., all vibrational modes of the molecules and adsorbates) in each subsystem. The CH_4_ and H_2_ molecules and the adsorbates of S1 and S2 have 9, 1, 9, and 18 vibrational modes, respectively. Thus, *M* = 1, 2, …, 18 for subsystem 1 and *M* = 1, 2, …, 19 for subsystem 2. Finally, the $\left\{\epsilon_{i}^{\mathrm{CH}4+\mathrm{ad}1}\right\}$ and $\left\{n_{i}^{\mathrm{CH}4+\mathrm{ad}1}\right\}$, and $\left\{\epsilon_{i}^{H2+\mathrm{ad}2}\right\}$ and $\left\{n_{i}^{H2+\mathrm{ad}2}\right\}$ are obtained. Using Equations (15) and (16), the pseudo-eigenstructure for the whole system is obtained as $\left\{\epsilon_{i}^{\mathrm{sub}1},\;\epsilon_{i}^{\mathrm{sub}2}\right\}$ and $\left\{n_{i}^{\mathrm{sub}1},\;n_{i}^{\mathrm{sub}2}\right\}$. The initial state is chosen to be a second-order hypoequilibrium state [41], for which the probability distribution in each subsystem $\left\{p_{i}^{\mathrm{sub}1}\right\}$, $\left\{p_{i}^{\mathrm{sub}2}\right\}$ takes a canonical form, namely:

(25) $$p_{i}^{\mathrm{sub}1}\left(t_{0}\right)=P^{\mathrm{sub}1}\frac{n_{i}^{\mathrm{sub}1}\exp\left(-\epsilon_{i}^{\mathrm{sub}1}/k_{b}T\right)}{Z^{\mathrm{sub}1}},\;p_{i}^{\mathrm{sub}2}\left(t_{0}\right)=P^{\mathrm{sub}2}\frac{n_{i}^{\mathrm{sub}2}\exp\left(-\epsilon_{i}^{\mathrm{sub}2}/k_{b}T\right)}{Z^{\mathrm{sub}2}}$$

Here, T represents the hypoequilibrium temperature and is set to 1000 °C, while $P^{\mathrm{sub}1}$ and $P^{\mathrm{sub}2}$ represent the total probability of each subsystem and are set at 0.99999999 and 0.00000001, respectively. The appropriateness of an initial hypoequilibrium state is discussed in detail in Reference [41].

### 3.3. Non-Equilibrium State Evolution

**Figure 4:**
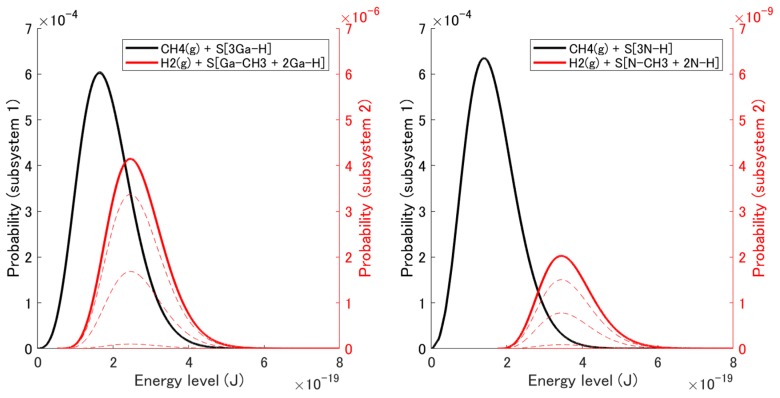
Probability distribution among the energy eigenlevels for the adsorption reaction in (0001) and (000−1). The bold solid lines correspond to the stable equilibrium state, while the narrow dashed lines correspond to intermediate states during the relaxation.

**Figure 5:**
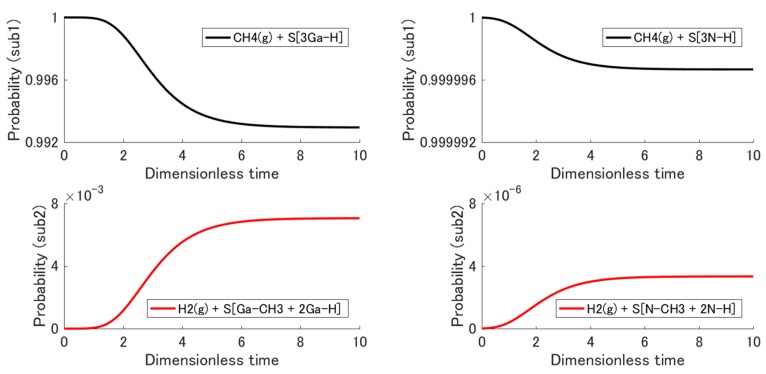
Evolution of the total probability of each subsystem for the adsorption reaction in (0001) and (000−1); that of subsystem 2 corresponds to the adsorption probability of CH_4_.

The non-equilibrium state evolution obtained from the initial state to the stable equilibrium state is discussed within the kinetics of the state trajectory, i.e., the dimensionless time evolution. Figure 4 shows the probability distribution $\left\{p_{i}^{\mathrm{sub}1},\;p_{i}^{\mathrm{sub}2}\right\}$ among the energy eigenlevels $\left\{\epsilon_{i}^{\mathrm{sub}1},\;\epsilon_{i}^{\mathrm{sub}2}\right\}$ for intermediate states during the relaxation (narrow dashed lines) and the final stable equilibrium state (bold solid lines). The black curves are the distributions for subsystem 1 (i.e., the reactants), and the red ones are those for subsystem 2 (i.e., the products). Note that the order of magnitude of each vertical axis is different from the other. Noting that the eigenlevels of subsystem 2 are higher than those of subsystem 1, one can see that the probability flows only slightly from subsystem 1 to subsystem 2. This is because of the gain in system entropy. In Section 2.2, the adsorption behavior is discussed using the chemical potential (i.e., free energy) of the molecule, which is just the macroscopic property (i.e., the expected value from the microscopic states) per molecule. This approach is useful for a qualitative discussion of which structure is more dominant. However, the SEAQT approach is essential for a detailed, quantitative discussion of the minor structure (CH_4_ adsorbed structure) because the chemical potential does not have detailed information about the small number of molecules different from the average ones. Only a few CH_4_ molecules are adsorbed onto the surface in the present case. Figure 5 shows the evolution of the total probability of each subsystem, $\sum p_{i}^{\mathrm{sub}1}\left(t\right)$ and $\sum p_{i}^{\mathrm{sub}2}\left(t\right)$. The adsorption probability, i.e., $P_{ad}\equiv \sum p_{i}^{\mathrm{sub}2}\left(t\right)$, (red line) reached 7.06 × 10^−3^ in (0001), and 3.32 × 10^−6^ in (000−1) at equilibrium. This difference in probabilities is remarkable and should influence the orientation dependence of the C impurity concentration, which will be discussed in Section 4.

**Figure 6:**
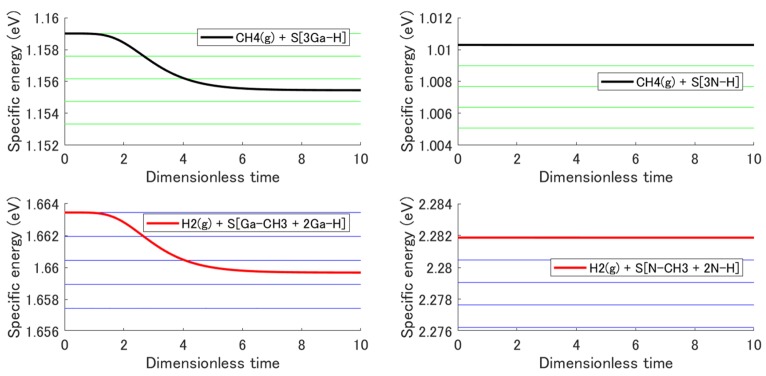
Evolution of the specific energy of each subsystem for the adsorption reaction in (0001) and (000−1). The green and blue horizontal lines correspond (going from top to bottom) to the specific energies at 1000, 999, 998, 997, and 996 °C.

In the present model, the system satisfies energy conservation. This leads to a decrease in system temperature due to the endothermic reaction. In a real system, the high thermal conductivity of the crystal keeps the temperature of the surface system constant. Therefore, the model must, in general, include an interaction with a heat reservoir. However, the heat reservoir is not needed if the temperature change is small enough. Figure 6 shows the evolution of the specific energy of each subsystem (the bold black and red lines) given by:

(26) $$\frac{\sum p_{i}^{\mathrm{sub}1}\left(t\right)\epsilon_{i}^{\mathrm{sub}1}}{\sum p_{i}^{\mathrm{sub}1}\left(t\right)},\;\frac{\sum p_{i}^{\mathrm{sub}2}\left(t\right)\epsilon_{i}^{\mathrm{sub}2}}{\sum p_{i}^{\mathrm{sub}2}\left(t\right)}$$

As can be seen, this energy decreases with system temperature. The horizontal narrow green and blue lines correspond to the specific energies at 1000, 999, 998, 997, and 996 °C from top to bottom, respectively. By comparing the specific energy evolution with these horizontal lines, one can observe that the temperature decrease was less than 3 °C in (0001) and approximately zero in (000−1).

## 4. Impurity Concentration

**Figure 7:**
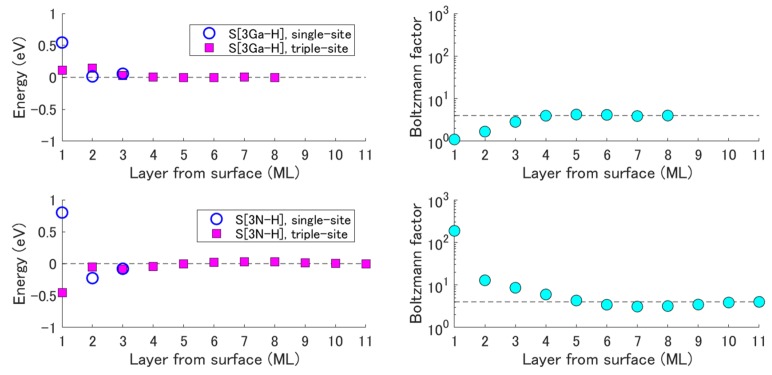
Depth profiles of the C impurity energy in (0001) and (000−1) as reported in Reference [51]. Weighted sum of Boltzmann factors (i.e., the sum of the triple one for the triple-site and the one for the single-site) at 1000 °C calculated from the energies.

Using the adsorption probability of CH_4_ obtained in Section 3.3, the growth orientation dependence of the C impurity concentration is discussed here. In 2017, Kempisty et al. reported the depth profiles of the C impurity energy in (0001) and (000−1), i.e., the comparison of the total energies of two-surface slab models, where the one nitrogen atom at the selected layer near the surface (i.e., the 1st, 2nd, …, 8th, or 10th atomic layers from the surface) is substituted by a carbon atom [49]. Figure 7 shows the result of Reference [51] for 3Ga-H and 3N-H surfaces and the Boltzmann factors, $\exp\left(-E/k_{B}T\right)$, at 1000 °C calculated from these energies. As can be seen, the C atoms at the fourth layer onwards from the surface were almost in the same situation as those in the bulk (i.e., layers deep enough). However, in (000−1), the C atoms at the second and third layers were somewhat different and the one at the first layer was greatly different from those in the bulk, which should influence the C impurity incorporation. Thus, here, only the difference in the first layer is considered for the C impurity incorporation model. 

If the same amount of the C atom were supplied to the surface in (0001) and (000−1), the amount of the C atom incorporated into the first layer would be proportional to the Boltzmann factor. As discussed in Section 3.3, in fact, the amount of the C atom supplied depends on the growth orientation. Therefore, the C impurity concentration $c_{C}$ at the first layer is also proportional to the adsorption probability:

(27) $$c_{C}\propto P_{\mathrm{ad}}\left(3\exp\left(-\frac{E_{\mathrm{triple}}}{k_{B}T}\right)+\exp\left(-\frac{E_{\sin\mathrm{gle}}}{k_{B}T}\right)\right)$$

Here, $P_{ad}$ is the CH_4_ adsorption probability. Note that there are two kinds of sites with different stability in the (2 × 2) area: three triple sites and one single site (see Reference [51]). Subsequently, these C atoms are incorporated into deeper layers via a non-equilibrium process, which means that the $c_{C}$ at the first layer almost decides the $c_{C}$ at the bulk. Therefore, the growth orientation dependence of the C impurity concentration at 1000 °C is estimated as follows:

(28) $$\frac{c_{C}^{\left(0001\right)}}{c_{C}^{\left(000-1\right)}}=\frac{P_{\mathrm{ad}}^{\left(0001\right)}}{P_{\mathrm{ad}}^{\left(000-1\right)}}\times \frac{\left[3\exp\left(-\frac{E_{\mathrm{triple}}^{\left(0001\right)}}{k_{B}T}\right)+\exp\left(-\frac{E_{\sin\mathrm{gle}}^{\left(0001\right)}}{k_{B}T}\right)\right]}{\left[3\exp\left(-\frac{E_{\mathrm{triple}}^{\left(000-1\right)}}{k_{B}T}\right)+\exp\left(-\frac{E_{\sin\mathrm{gle}}^{\left(000-1\right)}}{k_{B}T}\right)\right]}\;=\frac{7.06\times 10^{-3}}{3.32\times 10^{-6}}\times \frac{\left[3\exp\left(-\frac{0.111\mathrm{eV}}{k_{B}T}\right)+\exp\left(-\frac{0.545\mathrm{eV}}{k_{B}T}\right)\right]}{\left[3\exp\left(-\frac{-0.453\mathrm{eV}}{k_{B}T}\right)+\exp\left(-\frac{0.800\mathrm{eV}}{k_{B}T}\right)\right]}\;=\left(2.12\times 10^{3}\right)\times \left(5.89\times 10^{-3}\right)=1.25\times 10^{1}$$

The (000−1) growth orientation had the lower CH_4_ adsorption probability and the higher stability of the C impurity in the sub-surface than the (0001) growth orientation. As the result of our theoretical model, the C impurity concentration of GaN film grown in (000−1) was lower than that in (0001) by one order of magnitude. In experiments [52], the C impurity in (000−1) was also lower than that in (0001) by one order of magnitude or more, although it depended on the growth conditions. The model-estimated value, thus, agrees well quantitatively with the experimental one.

## 5. Conclusions

An sequential analysis based on first principles calculation was performed to theoretically estimate the C impurity concentration in GaN(0001) and (000−1) growth case. At the 3Ga-H (2 × 2) surface for (0001) and the 3N-H (2 × 2) surface for (000−1), which appear during the MOVPE process, where the CH_4_ adsorption structures are found to be those with the CH_3_ admolecule substituting the H adatom. In (0001), the stabilization of the structure proceeded to the CH_2_ (bridge-site) admolecule structure and to the CH (3H-site) admolecule structure. In addition, the SEAQT adsorption model is essential for a detailed discussion of the minor structure (i.e., the adsorption probability of the impurity atom) although the major structure (i.e., the stable surface reconstruction) can be discussed via a surface formation energies comparison, in which the macroscopic property as the expected value from the microscopic picture is used. In other words, the expected value (i.e., macroscopic property) can not be representative of the minor molecules, which play an important role in the impurity adsorption process. From the SEAQT analysis, a non-equilibrium state evolution of the adsorption process was obtained, and the adsorption probability was found to be 7.06 × 10^−3^ in (0001) and 3.32 × 10^−6^ in (000−1) at 1000 °C. A C impurity incorporation model was proposed here in which it was assumed that the C impurity concentration was proportional to the CH_4_ adsorption probability and to the Boltzmann factor calculated from the energy of the C impurity at the first surface layer [51]. As a result of this model, the ratio of the C impurity concentration in (0001) to that in (000−1), $c_{C}^{\left(0001\right)}/c_{C}^{\left(000-1\right)}$, was $1.25\times 10^{1}$. This estimate agrees well with experimental results [52]. Therefore, the feasibility of estimating the growth orientation dependence of the C impurity concentration using our sequential analysis approach was confirmed. That is, both the stability in crystal and the adsorption amount of the impurity, which depend on surface reconstruction, have to be considered for the quantitative estimation. For future work, the prediction could be improved by considering other possible carbon source molecules (e.g., Ga(CH_3_), Ga(CH_3_)_3_, C_2_H_4_, etc. [3,53]) or the mixed ratio of the most stable and metastable reconstructions.
